# Effects of combined maternal administration with alpha-ketoglutarate (AKG) and *β*-hydroxy-*β*-methylbutyrate (HMB) on prenatal programming of skeletal properties in the offspring

**DOI:** 10.1186/1743-7075-9-39

**Published:** 2012-05-11

**Authors:** Marcin R Tatara, Witold Krupski, Barbara Tymczyna, Tadeusz Studziński

**Affiliations:** 1 Department of Animal Physiology, Faculty of Veterinary Medicine, University of Life Sciences in Lublin, ul. Akademicka 12, 20-950, Lublin, Poland; 2 II Department of Radiology, Medical University of Lublin, ul. Staszica 16, 20-081, Lublin, Poland; 3 Department of Conservative Dentistry and Endodontics, Medical University of Lublin, ul. Karmelicka 7, 20-081, Lublin, Poland; 4 Department of Animal Physiology, Studium Generale Sandomiriense, ul. Krakowska 26, 27-600, Sandomierz, Poland

**Keywords:** Alpha-ketoglutarate, *β*-hydroxy-*β*-methylbutyrate, Prenatal programming, Skeletal system, Somatotrophic axis

## Abstract

**Background:**

Nutritional manipulations during fetal growth may induce long-term metabolic effects in postnatal life. The aim of the study was to test whether combined treatment of pregnant sows with alpha-ketoglutarate and *β*-hydroxy-*β*-methylbutyrate induces additive long-term effects on skeletal system properties in the offspring.

**Methods:**

The study was performed on 290 pigs obtained from 24 sows divided into 4 equal groups and subjected to experimental treatment during two weeks before delivery. The first group consisted of control sows, while the second group received alpha-ketoglutarate. The third group was treated with *β*-hydroxy-*β*-methylbutyrate and the fourth group underwent combined administration of alpha-ketoglutarate and *β*-hydroxy-*β*-methylbutyrate. Piglets obtained from sows were reared until slaughter age to perform morphometric, densitometric and mechanical analyses of femur. Serum evaluations of growth hormone, insulin-like growth factor-1, bone-specific alkaline phosphatase and osteocalcin were performed in newborns and 90-day old piglets; additionally, plasma amino acid concentration was measured in newborns.

**Results:**

Maternal treatment with alpha-ketoglutarate and *β*-hydroxy-*β*-methylbutyrate significantly reduced fattening time and increased birth body weight, daily body weight gain, bone weight, volumetric bone mineral density, geometrical parameters and mechanical endurance of femur. These effects were associated with increased serum concentrations of growth hormone, insulin-like growth factor-1, bone-specific alkaline phosphatase and osteocalcin. Furthermore, alpha-ketoglutarate and *β*-hydroxy-*β*-methylbutyrate administered solely or in combination significantly increased plasma level of 19 amino acids.

**Conclusions:**

Hormonal and amino acid evaluations in pigs indicate additive effects of AKG and HMB on systemic growth and development; however, determination of bone properties has not shown such phenomenon.

## Background

Osteoporosis is a metabolic disorder of skeletal system characterized by low bone mass and microarchitectural deterioration of bony tissue with consequent increased risk of fracture. Bone mass of an individual in later adult life depends upon the peak attained during skeletal growth and the subsequent rate of bone loss. Higher bone mass obtained during growth leads to lowered risk of osteoporosis development in the elderly, as well as following fractures. Thus, effective osteoporosis prevention might be aimed at increasing the peak bone mass (PBM) attained at the time of skeletal maturity [1]. Even though evidence suggesting that PBM is inherited, current genetic markers are able to explain only a small proportion of the variation in individual bone mass and fracture risk [2,3]. Approximately 20-30 % of the variation in PBM is determined by environmental factors such as nutrition and may be modified to obtain optimal bone mineral density, and morphological and mechanical properties of skeletal system [1]. Undernutrition and other adverse influences such as stress arising in fetal life or during neonatal life may have long-term effects on body structure, physiology, and metabolism. Its negative effects include disturbed amino acid and protein metabolism, altered gene expression, reduced cell numbers, imbalance between cell types, altered organ structure as well as changes in a pattern of hormonal release and tissue sensitivity to these hormones [4,5]. Epidemiological data obtained in humans have shown poor intrauterine growth and development as well as low birth weight as important factors associated with incidence of osteoporosis, hypertension, cardiovascular disease, glucose intolerance, insulin resistance, type II diabetes, dyslipidemia, obesity, schizophrenia, depression, and reproductive disorders [5-11]. Contrary to these data, experimental studies in animals and humans have shown that improved nutritional supply during critical stages of intrauterine and neonatal life characterized by very high sensitivity to metabolic, hormonal and nutritional factors may induce long-term beneficial effects on growth, development and health status [12-17]. The mechanisms responsible for stimulatory or inhibitory influence on development and growth of organisms at sensitive periods of prenatal and neonatal life, both in humans and animals, have long-term consequences in structure and functions of cells, tissues, organs and systems, inducing phenomenon termed “developmental programming” [18-20].

Alpha-ketoglutarate (AKG) is a molecule determining overall rate of the citric acid cycle. Reductive amination of AKG in perivein hepatocytes leads to glutamate synthesis that is converted to glutamine – conditionally essential amino acid which serves as the precursor of non-essential amino acids such as proline and arginine. Glutamate and glutamine are efficient donors of amine group in amination processes [21,22]. Both glutamine and glutamate are important sources of oxidative fuel for placenta during pregnancy, influencing fetal development [4]. In studies on animals, AKG administration has induced positive effects on skeletal development and homeostasis maintenance [23-25]. Furthermore, administration with AKG during neonatal life in sheep and pigs has induced beneficial effects on programming of skeletal system development in relation to bone mineral density, morphological and mechanical properties [12,14-16].

*β*-hydroxy-*β*-methylbutyrate (HMB) is a metabolite of an essential amino acid leucine and is produced through metabolism of alpha-ketoisocaproate (KIC). Approximately 5 % of leucine metabolism leads to endogenous synthesis of HMB that is converted to *β*-hydroxy-*β*-methylglutaryl coenzyme A (HMG-CoA) [26,27]. Studies with pregnant rats and their offspring exposed to experimental diet during prenatal life *in utero* have shown nutritional ability to program of the activity of HMG-CoA reductase – the enzyme responsible for conversion of HMG-CoA into mevalonate and following cholesterol synthesis [28]. *β*-hydroxy-*β*-methylbutyrate treatment in humans subjected to exercise resulted in increased muscle mass accretion associated with inhibition of muscle proteolysis [29]. Studies on rats and humans have shown that dietary supplementation with HMB solely or in combination with arginine and glutamine results in increased collagen deposition [30]. Furthermore, prenatal and neonatal exposure of pigs and sheep to HMB treatment has induced long-term beneficial effects on bone mineral density, as well as morphological and mechanical properties of skeletal system investigated at slaughter age [14,17].

The aim of the study was to test the hypothesis that treatment of pregnant sows during two last weeks of gestation with combined AKG and HMB may induce additive long-term effects on development of the skeletal system of the offspring. To achieve this aim, skeletal system properties of the offspring were investigated at slaughter age by determination of the volumetric bone mineral density, geometrical and mechanical properties of femur. Serum bone formation markers, concentrations of growth hormone (GH) and insulin-like growth factor-1 (IGF-1), as well as free amino acid concentrations in the plasma of the offspring were determined.

## Methods

The experimental procedures used throughout this study were approved by The Local Ethics Committee on Animal Experimentation of University of Life Sciences in Lublin, Poland.

## Experimental design of the study

The study was performed on 290 newborn pigs obtained from 24 sows of Polish Landrace (PL) breed as a result of physiological partum. All pregnant sows were divided into 4 groups and kept under identical breeding and environmental conditions for the whole period of experiment. The first group consisted of control sows (N = 6) treated with placebo (CaCO_3_ dissolved in saline at the daily dosage of 0.05 g/kg of body weight), while the second group of sows (N = 6) received alpha-ketoglutaric acid (AKG group) as a water solution buffered to neutral pH with the use of NaOH. The third group of sows (*N* = 6; HMB group) was treated with calcium salt of *β*-hydroxy-*β*-methylbutyric acid (CaHMB dissolved in saline) at the daily dosage of 0.05 g/kg of body weight. Sows (N = 6; AH group) that underwent the combined administration with alpha-ketoglutarate and *β*-hydroxy-*β*-methylbutyrate at the same dosages as in the AKG and HMB groups belonged to the fourth group. Throughout the last two weeks of gestation, the pregnant sows were administered *per os* with *placebo*, AKG and HMB daily during the morning meal. During the whole period of pregnancy and lactation all sows were fed twice a day at 0800 a.m. and 1600 p.m. with well balanced diet supplied in equal doses for all animals. The piglets obtained from sows were assigned to the control group (*N* = 71), AKG group (N = 73), HMB group (*N* = 72) and AH group (N = 74), accordingly with the treatment of their mothers. The pigs included in the different groups of the study were sired by the same boar. The body weights of the newborn piglets were determined postpartum and blood samples were immediately taken from the subclavian vein of unsuckled piglets for further analysis. Mortality of piglets (mainly due to squeezing by mothers or stress related to blood collection) was similar in each group and reached 10.7 % (31 piglets) in total. At the age of three days of life, all male piglets were castrated. The piglets from the control and experimental groups were kept with sows until the weaning at four weeks of life. During the whole period of the study, the piglets from all groups had free *ad libitum* access to fresh water and identical feed prepared according to the stage of the production cycle (Table 1). At the age of approximately 6 months, the offspring obtained from sows were slaughtered and left femur was isolated for further analyses. Finally, the control, AKG, HMB and AH groups consisted of 65 (36 females and 29 males), 63 (28 f and 35 m), 65 (30 f and 35 m) and 66 (32 f and 34 m) pigs, respectively.

**Table 1:** Composition of diet administered to pigs during the study

	Sows	14–27 day of life	28–42 day of life	7–12 week of life	13–19 week of life	From 20 week of life
**Ingredient**						
Wheat middlings (%)	35.0	47.0	45.0	40.0	30.0	25.0
Barley middlings (%)	35.0	20.0	36.0	35.5	30.0	30.0
Soybean meal (%)	15.0	18.0	10.0	17.5	15.0	10.0
Rye middlings (%)	10.5	–	–	–	22.0	32.5
Sauen-profi^1^* (%)	3.5	–	–	–	–	–
Ferkel-profi^2^* (%)	–	4.0	4.0	4.0	–	–
Josamin^3^* (%)	–	–	–	–	3.0	2.5
Megajoule* (%)	–	5.0	2.0	2.5	–	–
Cytromin duo* (%)	0.5	1.0	1.0	0.5	–	–
Trilac* (%)	–	5.0	2.0	–	–	–
Limestone (%)	0.3	–	–	–	–	–
Soybean oil (%)	0.7	–	–	–	–	–
**Composition**						
Dry matter (%)	88.45	89.32	88.81	88.64	88.30	87.25
Crude protein (%)	16.35	19.31	15.53	17.77	16.80	15.18
ME (MJ/kg)	12.96	13.21	12.89	12.94	12.99	13.07
Crude fibre (%)	3.56	3.41	3.43	3.68	3.50	3.34
Crude fat (%)	2.35	3.06	2.29	2.37	1.66	1.68
Ash (%)	5.43	6.10	5.50	5.70	4.76	4.22
Starch (%)	45.82	40.85	46.66	43.98	46.72	49.42
Ca (%)	0.81	0.84	0.80	0.81	0.75	0.63
P (%)	0.60	0.58	0.53	0.56	0.47	0.44
Available P (%)	0.16	0.15	0.15	0.15	0.14	0.13
Na (%)	0.17	0.22	0.21	0.21	0.17	0.15
Mg (%)	0.17	0.19	0.19	0.19	0.19	0.17
Carbohydrates (%)	3.90	4.98	4.85	4.40	3.54	3.50
Lys (%)	0.91	1.31	1.04	1.20	0.99	0.83
Met (%)	0.28	0.44	0.38	0.40	0.31	0.28
Met + Cys (%)	0.59	0.78	0.69	0.74	0.63	0.57
Trp (%)	0.20	0.27	0.21	0.23	0.20	0.18
Thr (%)	0.58	0.84	0.68	0.78	0.63	0.55
Vitamin A (IE)	18,000	65,000	38,000	30,000	12,000	10,000
Vitamin D_3_ (IE)	1,500	7,800	4,320	3,650	1,980	1,650
Vitamin E (mg)	90	307	202	145	180	150
Vitamin C (mg)	30.0	38.0	35.0	33.0	–	–
Vitamin K (mg)	2.1	2.5	2.5	2.5	1.40	1.20
Vitamin B_1_ (mg)	2.1	2.5	2.5	2.5	1.40	1.20
Vitamin B_2_ (mg)	4.4	6.5	6.5	6.5	4.24	3.52
Vitamin B_6_ (mg)	4.2	5.0	5.0	5.0	4.2	3.5
Vitamin B_12_ (μg)	14.0	38.0	35.0	35.0	35.0	30.0

^*1*^The vitamin-mineral concentrate contained per 1 kg of diet: L-lysine 6.0 %, Methionine 1.0 %, Threonine 0.5 %, Ca 20.0 %, P 7.5 %, Na 5.0 %, Mg 2.5 %, vitamin A 600,000 IU, vitamin D_3_ 50,000 IU, vitamin E 3,000 mg, vitanin C 1,000 mg, vitamin K_3_ 100 mg, vitamin B_1_ 90 mg, vitamin B_2_ 200 mg, vitamin B_6_ 180, vitamin B_12_ 1,330 μg, niacin 1,200, pantothenic acid 660 mg, folic acid 50 mg, choline chloride 15,000 mg, biotin 4,000 μg, Cu 875 mg, Zn 5,250 mg, Mn 3,000 mg, Fe 4,000 mg, J 75 mg, Co 10 mg, Se 10 mg.

^*2*^The vitamin-mineral concentrate contained per 1 kg of diet: L-lysine 10.0 %, Methionine 3.3 %, Threonine 4.2 %, Tryptophan 0.2 %, Ca 15.0 %, P 4.0 %, Na 4.5 %, vitamin A 500,000 IU, vitamin D_3_ 50,000 IU, vitamin E 3,300 mg, vitanin C 4,000 mg, vitamin K_3_ 125 mg, vitamin B_1_ 70 mg, vitamin B_2_ 150 mg, vitamin B_6_ 130, vitamin B_12_ 1,700 μg, niacin 1,700, pantothenic acid 500 mg, folic acid 40 mg, choline chloride 17,500 mg, biotin 6,000 μg, phytase 12,500 U, Cu 400 mg, Zn 1,000 mg, Mn 500 mg, Fe 800 mg, J 55 mg, Co 10 mg, Se 10 mg.

^*3*^The vitamin-mineral concentrate contained per 1 kg of diet: L-lysine 8.0 %, Methionine 2.0 %, Threonine 2.0 %, Ca 21.0 %, P 3.0 %, Na 5.0 %, Mg 2.0 %, vitamin A 400,000 IU, vitamin D_3_ 66,000 IU, vitamin E 6,000 mg, vitamin K_3_ 75 mg, vitamin B_1_ 70 mg, vitamin B_2_ 150 mg, vitamin B_6_ 130, vitamin B_12_ 1,000 μg, niacin 900, pantothenic acid 500 mg, folic acid 40 mg, choline chloride 12,000 mg, biotin 2,500 μg, phytase 10,000 U, Cu 160 mg, Zn 660 mg, Mn 160 mg, Fe 748 mg, J 50 mg, Co 11 mg, Se 13 mg.

*Josera Erbacher GmbH & Co., Kleinheubach, Germany.

## Blood collection and biochemical analyses in piglets

Blood samples were collected from unsuckled and 90-days old piglets for serum and plasma evaluations. The analyzed samples were collected from one male and one female form each litter, with the closest body weight values to mean body weight of the whole litter. The obtained samples were stored at –25°C until biochemical analyses. Bone-specific alkaline phosphatase (BAP) activity in serum of newborn piglets was performed with the use of an enzyme immunoassay (METRA BAP EIA kit, Quidel Corp., San Diego, CA, USA). The measurement of BAP concentration in serum was performed in 90-day old pigs using OCTEIA Ostase® BAP immunoenzymometric assay (Immunodiagnostic Systems Ltd., Boldon, Tyne and Wear, UK). Quantitative determination of osteocalcin in serum was performed by ELISA method (Human Osteocalcin ELISA, Diagnostic Systems Laboratories, Inc., Webster, TX, USA). Growth hormone (GH) concentration in serum of piglets was assessed with the use of Porcine Growth Hormone Enzyme-Linked Immunosorbant Assay – ELISA (Diagnostic Systems Laboratories, Inc., Webster, TX, USA). Serum concentration of insulin-like growth factor-1 (IGF-1) was determined using the commercial immunoenzymometric assay (IEMA) for the quantitative determination of IGF-1 in serum or plasma (OCTEIA IGF-1, Immunodiagnostic Systems Ltd., Boldon, Tyne and Wear, UK). The results of biochemical bone metabolism markers and hormonal analyses were obtained with the use of Benchmark Plus microplate spectrophotometer supplied with Microplate Manager Software Version 5.2.1 (Bio-Rad Laboratories, Inc., Hercules, CA, USA).

Determination of free amino acid concentration in plasma of newborn piglets was performed with the use of ion-exchange chromatography (INGOS AAA-400 apparatus for automatic analysis of amino acids, Ingos Corp., Prague, Czech Republic). Amino acids were separated using analytic column OSTION LG FA 3 mm x 200 mm. For amino acid separation five lithium cytrate buffers (pH 2.9, 3.1, 3.35, 4.05 and 4.9, respectively) were used. The amino acids were derivatized with ninhydrin and their identification was performed on the basis of retention time in comparison to the standards, using photocell combined with a computer. The software MIKRO version 1.8.0 was used for amino acids evaluation (Ingos Corp., Prague, Czech Republic).

## Bone analysis

Left femur was isolated 24 hours after slaughter from males (*N* = 133) and females (*N* = 126) and cleaned from remaining soft tissues. Bone length and bone weight were measured and bone samples were kept at –25°C until further analyses. The volumetric bone mineral density (vBMD) was measured using quantitative computed tomography (QCT) technique in SOMATOM EMOTION SIEMENS apparatus (Siemens, Erlangen, Germany) equipped with Somaris/5 VB10B software. The volumetric BMD was determined for both the trabecular and cortical bone on 2-mm thick, cross-sectional, metaphyseal and diaphyseal QCT scans, respectively. The trabecular bone mineral density (Td) was assessed on measuring scan placed at 18.5 % of total femur length, proximal to the distal growth plate. The cortical bone mineral density (Cd) was determined from the scan of diaphysis at 52 % of femur length, measuring from the distal bone extremity.

Mechanical properties of the femur were determined after 4 hour thawing at room temperature using three-point bending test in INSTRON 4302 apparatus (Instron, Canton, MA, USA) linked with a computer, registering relationship between forces perpendicular to the longitudinal axis of the bone and the resulting displacement. The values of maximum elastic strength (Wy) and ultimate strength (Wf) of the femur were determined. The distance between bone supports was set at 40 % of total femur length. The measuring head loaded bone samples with a constant speed of 20 mm/min.

Geometrical properties of the femur were determined on the basis of measurements of horizontal and vertical diameters of the mid-diaphyseal cross-section of this bone. The values of the cross-sectional area (A), the second moment of inertia (Ix), the mean relative wall thickness (MRWT) and the cortical index (CI) were calculated [31-33].

### Lean meat content determination

The evaluation of lean meat content of carcass was performed accordingly with the SEUROP grading method [34] with the use of Capteur Gras/Maigre – Sydel (CGM) apparatus (Sydel, Lorient, France). The lean meat content of carcass was calculated automatically by the CGM apparatus using the following formula:

Y = 50.11930 – 0.62421X_1_ + 0.26979X_2_ where:

Y – the estimated percentage of lean meat in the carcass,

X_1_ – the thickness of the fat (including rind), between the third and fourth last ribs (11th or 12th intercostal space) at 6 cm of the dorsal midline, at a trajectory perpendicular to the rind (in mm),

X_2_ – the thickness of the *Longisimus dorsi* muscle (in mm), measured at the X_1_ position.

## Statistical analysis

All data are presented as means ± SEM. The data were found to be normally distributed in accordance with Kolomogorov-Smirnov test. The obtained results of the investigated parameters of serum, plasma and femur in females and males were compared with the use of non-paired Student’s *t* test and did not revealed sex-related differences. Thus, the differences between the groups were tested for statistical significance with the use of one-way ANOVA. Post hoc comparisons of the differences were performed using Duncan’s test. Differences showing *P*-value ≤ 0.05 were considered as statistically significant.

## Results

### Body weights and growth rate of pigs

Mean values of birth weight of piglets in AKG, HMB and AH groups were significantly increased by 16 %, 23 % and 27 %, respectively when compared to the control group (P < 0.01). Significantly higher birth weights of piglets were stated in HMB and AH groups when compared to the AKG group (P < 0.05). Final body weight values were significantly higher in AKG and AH groups when compared to the control and HMB groups (P ≤ 0.01). Fattening time of pigs to final body weight was significantly shortened by 25, 17 and 27 days in the AKG, HMB and AH groups when compared to the controls (P < 0.001). Statistically significant differences of this parameter were also found in AKG and AH groups when compared to HMB group (P ≤ 0.001). Significantly increased values of daily body weight gain were stated in AKG, HMB and AH groups when compared to the controls (P ≤ 0.001). Furthermore, statistically higher values of daily body weight gain was stated in AKG and AH groups when compared to the control pigs (P < 0.001). However, mean lean meat content was not significantly differentiated in all the investigated groups (P > 0.05; Table 2).

**Table 2:** **Body weight, fattening time, daily body weight gain and lean meat content in piglets from the Control group and the groups being under prenatal influence of alpha-ketoglutarate (AKG Group),*β*-hydroxy-*β*-methylbutyrate (HMB Group) or alpha-ketoglutarate and*β*-hydroxy-*β*-methylbutyrate (AH Group)**

Investigated parameter	Control Group	AKG Group	HMB Group	AH Group
	(n = 65)	(n = 63)	(n = 65)	(n = 66)
Birth weight (g)	1315 ^a^	1525 ^b^	1622 ^c^	1675 ^c^
	± 41	± 31	± 36	± 39
Final body weight (kg)	102.8 ^a^	107.2 ^b^	101.2 ^a^	108.8 ^b^
	± 1.2	± 1.4	± 1.4	± 1.3
Fattening time (days)	203.8 ^a^	178.6 ^b^	186.8 ^c^	177.1 ^b^
	± 2.5	± 1.8	± 1.7	± 0.8
Daily body weight gain (g)	508.6 ^a^	602.5 ^b^	544.4 ^c^	615.2 ^b^
	± 7.7	± 8.5	± 8.9	± 8.2
Lean meat content (%)	51.92	52.42	51.86	52.10
	± 0.33	± 0.41	± 0.41	± 0.40

Values are means ± SEM.

^abc^Mean values that do not share common superscript letter in the same row differ significantly for P < 0.05 as compared by multiple range Duncan’s test.

### Free amino acid concentrations in plasma of newborn piglets

Results of free amino acid concentrations in plasma of newborn piglets are shown in Table 3. Alpha-ketoglutarate administration to pregnant sows has significantly increased plasma concentration of aspartate, serine, glutamine, glycine, alanine, valine, ornithine, lysine and arginine in newborns when compared to the control group (P < 0.05). *β*-hydroxy-*β*-methylbutyrate administration to sows during two weeks before the partum induced significantly higher concentration of glutamine, glycine, valine, tyrosine in plasma of the newborns when compared to the control group (P < 0.05). Plasma concentration of cystine was significantly decreased in newborn piglets assigned to HMB group when compared to the controls (P < 0.001). Combined administration of AKG and HMB in the AH group of sows induced significantly higher concentration of aspartate, threonine, serine, glutamine, proline, glycine, citruline, valine, cystine, methionine, isoleucine, leucine, tyrosine, phenylalanine, ornithine, lysine, histidine and arginine in blood plasma when compared to the control newborns (P < 0.05).

**Table 3:** **Free amino acids concentrations (nmol/mL) in plasma of newborn piglets from the Control group and the groups being under prenatal influence of alpha-ketoglutarate (AKG Group),*β*-hydroxy-*β*-methylbutyrate (HMB Group) or alpha-ketoglutarate and*β*-hydroxy-*β*-methylbutyrate (AH Group)**

Amino acid	Control Group	AKG Group	HMB Group	AH Group
	(n = 12)	(n = 12)	(n = 12)	(n = 12)
Cysteic acid	11 ± 0.3	14 ± 1	13 ± 1	12 ± 1
Taurine	207 ± 11	195 ± 17	174 ± 18	202 ± 26
Aspartate	23 ± 1 ^a^	34 ± 3 ^b^	28 ± 2 ^ab^	34 ± 2 ^b^
Threonine	213 ± 23 ^a^	159 ± 15 ^a^	158 ± 10 ^a^	322 ± 26 ^b^
Serine	210 ± 4 ^a^	312 ± 23 ^b^	236 ± 16 ^a^	426 ± 20 ^c^
Glutamate	126 ± 14	136 ± 20	99 ± 12	141 ± 15
Glutamine	217 ± 11 ^a^	307 ± 20 ^b^	286 ± 33 ^b^	479 ± 21 ^c^
Proline	198 ± 25 ^a^	416 ± 35 ^b^	169 ± 18 ^a^	1045 ± 72 ^c^
Glycine	870 ± 37 ^a^	1420 ± 101 ^b^	1127 ± 74 ^c^	1474 ± 84 ^b^
Alanine	1098 ± 62 ^a^	1816 ± 153 ^b^	1138 ± 55 ^a^	1371 ± 82 ^a^
Citrulline	68 ± 3 ^a^	76 ± 5 ^a^	83 ± 6 ^a^	148 ± 9 ^b^
Valine	266 ± 11 ^a^	341 ± 23 ^b^	328 ± 19 ^b^	518 ± 24 ^c^
Cystine	47 ± 3 ^a^	58 ± 5 ^ab^	27 ± 3 ^c^	65 ± 4 ^b^
Methionine	15 ± 2 ^a^	26 ± 2 ^a^	19 ± 3 ^a^	85 ± 10 ^b^
Isoleucine	27 ± 4 ^a^	33 ± 4 ^a^	19 ± 2 ^a^	110 ± 8 ^b^
Leucine	83 ± 10 ^a^	96 ± 8 ^a^	95 ± 6 ^a^	340 ± 17 ^b^
Tyrosine	71 ± 6 ^a^	67 ± 5 ^a^	108 ± 7 ^b^	236 ± 19 ^c^
Phenylalanine	44 ± 5 ^a^	58 ± 4 ^a^	48 ± 4 ^a^	157 ± 11 ^b^
Tryptophan	37 ± 12	17 ± 5	17 ± 8	21 ± 2
Ornithine	71 ± 4 ^a^	104 ± 8 ^b^	83 ± 11 ^ab^	153 ± 14 ^c^
Lysine	268 ± 12 ^a^	394 ± 25 ^b^	280 ± 24 ^a^	655 ± 30 ^c^
Histidine	24 ± 5 ^a^	31 ± 3 ^a^	42 ± 3 ^a^	142 ± 11 ^b^
Arginine	81 ± 5 ^a^	121 ± 12 ^b^	105 ± 7 ^ab^	235 ± 21 ^c^

Values are means ± SEM.

^abc^Mean values that do not share common superscript letter in the same row differ significantly for P < 0.05 as compared by multiple range Duncan’s test.

### Concentrations of bone metabolism markers and hormones in serum of newborn and 90-day old piglets

Results of measurements of biochemical bone metabolism markers and hormones in serum of newborn and 90-day old piglets are presented in Tables 4 and 5. Serum concentration of osteocalcin was not significantly different in the experimental groups of newborns when compared to the control piglets (P > 0.05). At the age of 90 days of life, significantly increased serum concentration of octeocalcin was obtained in AH group when compared to the control, AKG and HMB groups (P ≤ 0.01). Bone alkaline phosphatase activity was significantly higher in newborns from AKG, HMB and AH groups when compared to the control piglets (P < 0.05). Serum concentration of BAP in 90-day old piglets reached significantly higher values in AKG and HMB groups when compared to the control and AH groups (P < 0.001). Furthermore, significantly higher concentration of BAP was stated in AH group when compared to the controls (P = 0.05). The mean serum GH concentration in the newborns from the HMB and AH groups reached significantly increased values by 55 % and 88 %, when compared to the control group (P < 0.01). Significantly higher serum GH concentration was stated in AH group of newborns when compared to the AKG group (P = 0.003). At the age of 90 days of life, serum concentration of GH was found to be higher in AH group when compared to the piglets from AKG group (P = 0.02). Significantly higher values of serum concentration of IGF-1 were found in all the experimental groups of newborns when compared to the control piglets (P < 0.05). Moreover, significantly higher values of IGF-1 concentration in serum were stated in AKG and AH groups when compared to the HMB group (P < 0.05). Significantly higher values of serum concentration of IGF-1 were stated in all the experimental groups of 90-day old piglets when compared to the control group (P < 0.05). Significantly higher values of IGF-1 concentration in serum were noted in AKG and AH groups when compared to the HMB group (P < 0.001). Significantly higher value of serum IGF-1 concentration was obtained in 90-day old pigs from AH group when compared to the AKG group (P = 0.006).

**Table 4:** **Serum concentration of biochemical markers of bone metabolism and hormones in newborn piglets from the Control group and the groups being under prenatal influence of alpha-ketoglutarate (AKG Group),*β*-hydroxy-*β*-methylbutyrate (HMB Group) or alpha-ketoglutarate and*β*-hydroxy-*β*-methylbutyrate (AH Group)**

Investigated parameter	Control Group	AKG Group	HMB Group	AH Group
	(n = 12)	(n = 12)	(n = 12)	(n = 12)
Osteocalcin (ng/mL)	24.0 ^ab^	28.5 ^a^	23.9 ^ab^	16.5 ^b^
	± 3.7	± 4.2	± 3.5	± 3.4
Bone alkaline phosphatase activity (U/L)	20.9 ^a^	28.8 ^b^	27.9 ^b^	26.4 ^b^
	± 1.4	± 1.6	± 1.3	± 1.9
Growth hormone (ng/mL)	18.1 ^a^	23.4 ^ab^	28.0 ^bc^	34.1 ^c^
	± 1.9	± 2.6	± 1.9	± 3.2
Insulin-like growth factor-1 (μg/L)	49.1 ^a^	97.9 ^b^	73.8 ^c^	113.2 ^b^
	± 4.8	± 9.5	± 2.9	± 10.8

Values are means ± SEM.

^abc^Mean values that do not share common superscript letter in the same row differ significantly for P < 0.05 as compared by multiple range Duncan’s test.

**Table 5:** **Serum concentration of biochemical markers of bone metabolism and hormones in 90-day old pigs from the Control group and the groups being under prenatal influence of alpha-ketoglutarate (AKG Group),*β*-hydroxy-*β*-methylbutyrate (HMB Group) or alpha-ketoglutarate and*β*-hydroxy-*β*-methylbutyrate (AH Group)**

Investigated parameter	Control Group	AKG Group	HMB Group	AH Group
	(n = 12)	(n = 12)	(n = 12)	(n = 12)
Osteocalcin (ng/mL)	4.8 ^a^	3.9 ^a^	4.4 ^a^	5.9 ^b^
	± 0.2	± 0.2	± 0.2	± 0.4
Bone alkaline phosphatase concentration (μg/L)	220 ^a^	345 ^b^	330 ^b^	242 ^c^
	± 4.4	± 13.3	± 7.1	± 6.5
Growth hormone (ng/mL)	3.4 ^ab^	2.6 ^a^	3.4 ^ab^	4.7 ^b^
	± 0.9	± 0.3	± 0.5	± 0.7
Insulin-like growth factor-1 (μg/L)	18.5 ^a^	63.5 ^b^	29.1 ^c^	86.4 ^d^
	± 1.9	± 3.5	± 3.7	± 9.4

Values are means ± SEM.

^abc^Mean values that do not share common superscript letter in the same row differ significantly for P < 0.05 as compared by multiple range Duncan’s test.

### Volumetric bone mineral density, mechanical and morphometric properties of femur in pigs at slaughter age

Results of volumetric bone mineral density in the trabecular and cortical bones, morphological properties, and mechanical properties of femur in pigs from the control and experimental groups are presented in Table 6. Bone weight was found to be significantly increased in all the experimental groups when compared to the control group (P < 0.001). Significantly increased mean value of this parameter was stated in the AH group when compared to the AKG group (P = 0.01). Length of femur reached significantly higher value in AH group when compared to all other groups (P ≤ 0.01). Mean value of Td was significantly higher in HMB group when compared to the control, AKG and AH groups (P < 0.001). Furthermore, the values of Td obtained in AKG and AH groups were significantly higher when compared to the controls (P < 0.001). Mean value of Cd was significantly higher in HMB group when compared to the control, AKG and AH groups (P < 0.05). The values of Cd obtained in AKG and AH groups were significantly higher when compared to the control group (P < 0.001). Cross-sectional area measured in the midshaft of femur reached significantly higher values in all experimental groups when compared to this parameter in the controls (P ≤ 0.001). Second moment of inertia of femur reached significantly higher values in the HMB and AH groups when compared to the control group (P ≤ 0.01). Moreover, the comparison of this parameter between AKG and AH groups revealed its higher value in the piglets which were under combined prenatal influence of AKG and HMB (P = 0.01). The determination of MRWT and CI of femur has shown significantly higher values of these parameters in the groups AKG and HMB when compared to the control and AH groups (P ≤ 0.01). Mechanical testing of femur revealed significant increase of maximum elastic strength in all the experimental groups when compared to the controls (P ≤ 0.01). Significantly higher values of Wy of femur was noted in the HMB group when compared to the AH group (P = 0.01). Ultimate strength of femur reached significantly higher values in all experimental groups when compared to the control group (P ≤ 0.01).

**Table 6:** **Volumetric bone mineral density, morphometric and mechanical parameters of femur in pigs at slaughter age from the Control group and the groups being under prenatal influence of alpha-ketoglutarate (AKG Group),*β*-hydroxy-*β*-methylbutyrate (HMB Group) or alpha-ketoglutarate and*β*-hydroxy-*β*-methylbutyrate (AH Group)**

Investigated parameter	Control Group	AKG Group	HMB Group	AH Group
	(n = 65)	(n = 63)	(n = 65)	(n = 66)
Bone weight (g)	300.1 ^a^	319.3 ^b^	324.8 ^bc^	333.7 ^c^
	± 4.1	± 3.5	± 3.5	± 4.0
Bone length (mm)	197.4 ^a^	197.7 ^a^	196.9 ^a^	201.0 ^b^
	± 1.0	± 1.1	± 1.0	± 0.8
Trabecular bone mineral density (g/cm^3^)	1.360 ^a^	1.414 ^b^	1.454 ^c^	1.410 ^b^
	± 0.005	± 0.006	± 0.007	± 0.005
Cortical bone mineral density (g/cm^3^)	2.387 ^a^	2.565 ^b^	2.603 ^c^	2.560 ^b^
	± 0.020	± 0.010	± 0.008	± 0.009
Cross-sectional area (mm^2^)	288.3 ^a^	312.9 ^b^	319.5 ^b^	320.8 ^b^
	± 6.8	± 3.9	± 4.9	± 4.7
Second moment of inertia (mm^4^)	17 536 ^a^	19 065 ^ab^	19 851 ^bc^	21 396 ^c^
	± 716	± 492	± 584	± 644
Mean relative wall thickness	0.601 ^a^	0.668 ^b^	0.703 ^b^	0.620 ^a^
	± 0.014	± 0.012	± 0.017	± 0.012
Cortical index	37.16 ^a^	39.70 ^b^	40.78 ^b^	37.95 ^a^
	± 0.536	± 0.433	± 0.578	± 0.467
Maximum elastic strength (N)	4066 ^a^	4854 ^bc^	5184 ^c^	4644 ^b^
	± 172	± 139	± 170	± 129
Ultimate strength (N)	5377 ^a^	5964 ^b^	6288 ^b^	6229 ^b^
	± 168	± 130	± 153	± 169

Values are means ± SEM.

^abc^Mean values that do not share common superscript letter in the same row differ significantly for P < 0.05 as compared by multiple range Duncan’s test.

## Discussion

This study has shown that prenatal administration with AKG and HMB, individual or in combination, increases body weight of newborn piglets. The most notable increases of body weight at birth were seen in piglets born by sows with combined treatment of AKG and HMB than in HMB only group and reached 27 % and 23 %, respectively, while this parameter was improved by only 16 % in the AKG group. Similar effects were obtained for fattening time and daily body weight gain. The most advantageous effects were stated in the AKG and AH groups where fattening time was reduced by 25 and 27 days, and the final body weight in these groups was increased by 4.3 % and 5.8 %, respectively. Even though final body weight in pigs was not influenced by prenatal administration with HMB, beneficial effects in this group was expressed as accelerated postnatal growth rate and improved the daily body weight gain. These results are in accordance with previous investigations on pig model where administration of AKG during last 24 days of pregnancy increased body weight of newborns by 29 % [35]. The 13 % difference between the scales of improved body weight of newborns in both these investigations may results form 10 day longer administration of AKG in previous studies, since the same daily dosage and form of AKG was used. In the other report on prenatal influence of AKG during the last 24 days of pregnancy, body weight in newborn and 14 day old piglets was improved by 29 % and 6 %, respectively. Unfortunately in that study, the offspring from sows receiving AKG were sacrificed at 2 weeks of life and constituted a short-term effect study, and so it was not possible to compare the results achieved there to our long-term effect results [36]. Twenty-one and 24-day long neonatal treatment of piglets with AKG at the daily dosages of 0.1 and 0.3 g/kg of body weight increased growth rate in 10- and 21-day old females, whilst in males opposite effects were obtained and lighter body weights were recorded at these developmental stages. However, administration with AKG has not induced long-term effects on final body weights of the animals slaughtered at the age of 169 days of life, indicating positive effects lasting only as long as it is being administered [16]. Investigations on HMB administration to sows during the last 2 weeks of pregnancy have showed birth body weight of male and female piglets increased by 21 % and 26 % when compared to the controls. Furthermore, as the consequence of prenatal influences of HMB, the fattening time to final body weight of the pigs was reduced by 11 days, and the body weights at slaughter in these pigs were not different from controls in both males and females [14]. In other studies on chicken embryos model, *in ovo* administration of HMB increased body weight of hatched and 10-day chicks by 3.3 % and 4.7 %, respectively [27]. Experiments performed on turkey embryos have also shown positive effects of *in ovo* feeding with HMB on body weight from hatch to 14 days of age when the study was finished [37]. Similarly to the outcome in our study, the only one available report on combined treatment with AKG and HMB in growing fundectomized pigs has not revealed additive effects of these metabolites on final body weight that reached comparable values in 8-month old animals receiving AKG or HMB only. However, the final body weights of pigs growing over 6 months without the fundic part of the stomach and fed AKG, HMB or AKG and HMB *per os* were increased double when compared to the fundectomized group receiving placebo [38].

Effects of prenatal administration with AKG and HMB on systemic growth and development of piglets in postnatal life observed in our study were associated with increased plasma concentration of free amino acids. The most prominent effects of prenatal dietary manipulations on amino acid metabolism were observed in the group being under combined influence of AKG and HMB. In this group, 18 amino acids such as aspartate, threonine, serine, glutamine, proline, glycine, citrulline, valine, cystine, methionine, isoleucine, leucine, tyrosine, phenylalanine, ornithine, lysine, histidine and arginine were found to be increased in plasma of newborns in comparison to the control group. In case of threonine, serine, glutamine, proline, citrulline, valine, methionine, isoleucine, leucine, phenylalanine, ornithine, lysine, histidine and arginine, the obtained results indicate an additive anabolic effects of AKG and HMB on amino acid metabolism, since the amino acid plasma concentrations were significantly higher than in the groups receiving solely AKG or HMB. In the AKG group, concentrations of 10 amino acids such as aspartate, serine, glutamine, proline, glycine, alanine, valine, ornithine, lysine and arginine were elevated above the control values. Noteworthy is the fact that in the AH and AKG groups, the final body weights as well as daily body weight gain and fattening time reached the most desirable characteristics. In the HMB group, relatively moderate effects of the dietary manipulation on plasma amino acid concentration were observed and the level of glutamine, glycine, valine and tyrosine was increased in comparison to the control group. Results obtained in our study are in accordance with the previous investigations on weaned pigs where an enteral 6-hour continuous infusion of 930 μmol/kg/h of AKG improved net portal absorption rate of proline, leucine and lysine by 64 %, 49 % and 36 %, respectively. The concentrations of all these amino acids were increased in our study in newborn piglets being under individual or combined prenatal influence of AKG [39]. Moreover, in 17-week old turkeys, plasma concentrations of proline and leucine were elevated by 53 % and 25 % after 14-week long oral administration with AKG at the same dosage as in the current study [24]. Available data from studies on growing turkeys have shown that 15-week administration with HMB increased concentration of cysteic acid, glutamine, valine, aspartate, glutamate, proline, alanine, isoleucine, leucine and phenylalanine, whilst body weights were not different in comparison to control birds [40]. In studies on fundectomized pigs, HMB improved plasma concentration of methioine, threonine, valine, leucine, tyrosine, tryptophan and arginine, depending on blood sampling time since last oral dosage of HMB [41]. However, simultaneous administration with AKG and HMB improved concentration of histidine, methionine and threonine when evaluated in plasma of fundectomized 8-month old pigs subjected to 24-hour fasting [38].

The current study also indicates that positive effects of prenatal administration with AKG and HMB were associated with improved somatotrophic axis function due to increased serum levels of GH and IGF-1. In the newborns, GH level was increased in animals being under influence of HMB only or HMB introduced simultaneously with AKG, while IGF-1 reached higher values in all the investigated groups. In 90-day old piglets, GH was found to be increased only in the group born by the sows receiving combined treatment of AKG and HMB but IGF-1 concentration was found to be significantly elevated in all the experimental groups. These data suggest that higher serum concentration of IGF-1 in all the groups receiving experimental treatments may results from enhanced somatotrophic axis function and liver production of IGF-1 as well as locally produced IGF-1 and its autocrine or paracrine actions [42]. Recent experiments on rats performed by Gerlinger-Romero and colleagues (2011) have shown that chronic supplementation of HMB increases the activity of the GH/IGF-1 axis and induces increased content of IGF-1 mRNA in the liver [43]. It was shown in previous studies on humans that AKG administered as ornithine salt (OKG) in multiple trauma adult patients, who were highly catabolic and hypermetabolic, increases plasma levels of IGF-1 and GH by 41 % and 82 %. Similarly to the results in this study, OKG administration in those patients enhanced glutamine, proline and ornithine levels by 25 %, 39 % and 234 %, respectively [44]. In children suffering from growth retardation, 5-month long OKG administration (15 g/day) induced growth acceleration in association with increased plasma IGF-1 level. Moreover, these effects were related to significantly higher plasma glutamine concentration in children receiving AKG [45]. Similarly to studies on humans, elevated levels of GH and IGF-1 in newborn piglets from AKG-treated sows during the last 24 days of pregnancy were stated [36]. As opposite to studies on pigs, 14-day neonatal treatment with AKG in lambs has not induced effects on circulating IGF-1 level. However, 4 times lower dosage of AKG was administered to sheep than in the current study [12]. Investigations on animals with maternal and neonatal administration of HMB have also shown improved somatotrophic axis function. HMB treatment in pregnant sows for 2 weeks before the partum increased serum GH and IGF-1 levels by 38 % and 20 % in the offspring [14]. In lambs, oral administration with HMB during the first 21 days of life also increased GH and IGF-1 concentration by 70 % as determined at 3 weeks of life, while at the age of 130 days of life both these hormones reached similar values to those in controls [17]. Thus, it may be postulated that prenatal treatment with HMB may induce long-term effects on secretive function of the somatotrophic axis, while neonatal or postnatal administration improves temporary its function and brings short-term effects.

Next to the observed beneficial effects of prenatal influences of AKG and HMB on increased growth rate of pigs and hormonal and amino acid status, this study demonstrated improved morphometric, densitometric and mechanical properties of bones after the experimental treatments. Both these metabolites induced individually or in combination long-term anabolic effects on bone tissue metabolism which were expressed as increased values of bone formation marker – BAP in newborn and 90-day old piglets. Increased serum concentration of OC in 90-day old pigs in the AH group also confirmed continuous acceleration of bone formation processes resulting from the performed dietary manipulation in pregnant sows. However, the observed in this study difference of serum OC concentration between AKG and AH groups of newborns need further investigation to be explained. Among morphological traits, weight of femur was improved in all the experimental groups and the most readable effects were induced after exclusive or combined administration with HMB, while bone length was improved by nearly 2 % in case of the combined treatment. Improved weight of femur may results from both the observed increased vBMD and bone geometry. It is noteworthy that cross-sectional area was enhanced in all the experimental groups, while second moment of inertia reached higher values in the HMB and AH groups. As the result of improved periosteal and endoosteal bone deposition, MRWT and CI of femur were higher after exclusive treatment with AKG or HMB. Prenatal treatment with AKG and HMB increased also vBMD within both cancellous and compact compartments in all the experimental groups. It is surprising that the highest values of Td and Cd were stated in animals receiving HMB only, and the additive effect of AKG and HMB on bone mineralization was not induced. Evaluation of mechanical endurance of femur has not revealed additive effects of AKG and HMB; however, improved maximum elastic strength and ultimate strength were observed in all the experimental groups. The improved mechanical characteristic of femur observed in all the experimental groups seems to be result of improved bone geometry and vBMD since both these factors determine bone rigidity and mechanical endurance to acting forces [46-48]. The observed anabolic effects of AKG and HMB on skeleton in the current study are in accordance to the results obtained on the fundectomized pig model, where severe osteopenia development of femur and tibia was inhibited by a 6-month administration with these metabolites. In AKG-treated animals, significant increase of weight, length, bone mineral density (BMD), bone mineral content (BMC), cross-sectional area, second moment of inertia, mean relative wall thickness, cortical index, maximum elastic strength and ultimate strength of the bones was associated with improved serum concentration of IGF-1 and serum BAP activity when compared to the fundectomized group [49]. Results of long bone analysis in slaughter pigs treated during 21 and 24 days of neonatal life with AKG revealed its positive effects on length, Cd, Wy, Wf and Young’s modulus that was connected with elevated plasma estrogens level [16]. In studies on growing turkeys, 14-week long administration with AKG eliminated neurectomy-induced osteopenia of radius increasing its weight, vBMD, A, Ix, MRWT, Wy and Wf. These advantageous effects were combined with higher serum concentration of proline and leucine in comparison to placebo-treated birds [24]. In other studies on sheep, two week long neonatal treatment with AKG improved Td, Cd and Wf of femur as well as increasing weight, length, Cd, Wy and the moments of maximum elastic strength and ultimate strength [12,15]. Furthermore, it was shown that 7-week administration with OKG in growing female turkeys induces higher Td, Cd, Wy and Wf of tibia without any changes of bone geometry. However, increased synthesis of aspartate, proline, alanine, valine, leucine, isoleucine and ornithine was postulated to be responsible for enhanced skeletal properties [50]. Using fendectomized pig model, it was revealed that HMB inhibits fundectomy-induced osteopenia in axial skeleton (lumbar vertebrae) improving weight, total volume, mean vBMD, Td, calcium hydroxyapatite density of trabecular and cortical bone, BMD, BMC, ultimate force, ultimate stress, Young’s modulus, stiffness and work to the ultimate force point. Together with the beneficial influence of HMB on bone tissue and skeletal characteristic, the improved synthesis of threonine, glycine, alanine, valine, methionine, leucine, tyrosine, tryptophan and arginine was observed, confirming importance of amino acid metabolism for bone formation and skeletal homeostasis maintenance [41]. In studies on growing meat-type male turkeys, HMB administration from 6 to 20 week of life positively influenced geometry, vBMD and mechanical endurance of tibia. It was shown that improved Td, Cd, A, Ix, Wy and Wf of tibia was related to higher plasma concentration of cysteic acid, aspartate, glutamate, alanine, valine, leucine, isoleucine and phenylalanine [39]. Unfortunately, the comparison of the bone properties determined in this study on pigs from the AH group is not possible with other experimental data, since effects of combined treatment with AKG and HMB on bone tissue metabolism and skeletal system were not previously studied, either in animals or in humans.

Considering the results obtained in the current study, it should be emphasized that prenatal dietary manipulation with AKG and HMB influenced systemic development of pigs stimulating different physiological mechanism. It may be postulated that increased birth and final body weight, as well as accelerated systemic growth and development of pigs were induced via stimulated secretory function of the somatotrophic axis by AKG and HMB. This hypothesis is supported by the data showing that endocrine gland function can be programmed *in utero* at critical periods of prenatal development with metabolic pre-partum and post-partum consequences on the cellular, tissue and system levels [9]. It may be proposed that the improved skeletal properties in AKG and/or HMB treated animals result from positive prenatal programming of the somatotrophic axis, especially when one considers decisive role of GH and IGF-1 for skeletal growth, osteoblasts function, bone mineralization and calcium-phosphate homeostasis maintenance [51]. The other mechanism positively influencing prenatal development of piglets, their birth and final body weight, as well as daily body weight gain and bone tissue properties may result from improved amino acid metabolism. It must be underlined that plasma glutamine concentration was increased in newborns from all the experimental groups. Besides its building function in protein and polypeptides structures, glutamine - being a functional amino acid - determines key metabolic pathways necessary to provide optimal growth rate and immunity. It maximizes efficiency of feed utilization, enhances protein accretion and improves health status influencing effectives in animal production. Furthermore, glutamine, as the most abundant alpha-amino acid in skeletal muscles, regulates nutrient metabolism, gene expression and protein synthesis – the main factors influencing muscle mass and body weight [52]. Improved metabolism of glutamine would also provide benefit for skeletal formation, especially when one considers its role as the main substrate for synthesis of proline – the amino acid undergoing hydroxylation process to form hydroxyproline. Both proline and hydroxyproline are considered as integral constituents of collagen helix and its protectors against proteases. While proline and hydroxyproline contribute to two-thirds of the collagen structure, one-third is ascribed to glycine [53]. Except for proline concentration in the HMB group of newborns, both proline and glycine were increased in all the experimental groups. Thus, AKG- and HMB induced increase of the amount of collagen in bones of the newborns from experimental groups would provide steady advantage in bone mass of the skeleton maintained or increased during postnatal growth and development in relation to control animals. It can not be excluded that higher muscle mass since birth of piglets has also induced positive effects on skeleton through higher life-long tension and biomechanical stimulation of bones and their positive response in terms of enhanced mineralization, morphological traits and mechanical endurance. Better muscle function resulting from direct and indirect influences of the other amino acids (mostly leucine, isoleucine, valine, arginine and methionine) increased in AKG- and HMB-treated piglets would also be effective in relation to improved muscle-bone interactions [54]. Due to observed higher body weights since birth in all the experimental groups of pigs than in the control animals, positive effects in bones may results partially from heavier skeletal loading, since adaptive response of bone tissue to loading increases bone mass, size and mineral density [55,56].

In response to the question whether additive effects of maternal treatment with AKG and HMB on prenatal programming of growth, development and bone tissue metabolism exist, which arises from the current study, the complete explanation of this issue seems to be difficult. It must be underlined that in the AH group, the highest plasma concentration of 16 amino acids such as threonine, serine, glutamine, proline, citruline, valine, cysteine, methionine, isoleucine, leucine, tyrosine, phenylalanine, ornithine, lysine, histidine and arginine in comparison to all other groups indicates on additive effects of these metabolites in relation to prenatal programming of growth, development and bone tissue metabolism. The observed secretive response of the somatotrophic axis to combined maternal treatment with AKG and HMB and the highest concentration of GH and IGF-1 in the AH group confirm additive effects of these metabolites in relation to systemic growth and development. However, except for bone length, the evaluation of systemic growth parameters and bone properties in pigs at slaughter age has not shown additive effects of prenatal AKG and HMB application.

## Abbreviations

PBM, Peak bone mass; AKG, Alpha-ketoglutarate; HMB, β-hydroxy-β-methylbutyrate; KIC, Alpha-ketoisocaproate; HMG-CoA, β-hydroxy-β-methylglutaryl coenzyme A; GH, growth hormone; IGF-1, Insulin-like growth factor-1; BAP, Bone-specific alkaline phosphatase; OC, Osteocalcin; vBMD, Volumetric bone mineral density; QCT, Quantitative computed tomography; Td, Trabecular bone mineral density; Cd, Cortical bone mineral density; Wy, Maximum elastic strength; Wf, Ultimate strength; A, Cross-sectional area; Ix, Second moment of inertia; MRWT, Mean relative wall thickness; CI, Cortical index; OKG, Ornithine alpha-ketoglutarate; BMD, Bone mineral density; BMC, Bone mineral content.

## Competing interests

The authors of the manuscript have no competing interests.

## Authors’ contributions

MT, as the principal investigator, was responsible for the concept and experimental design of the study and supervised all stages of the experiment. MT was also responsible for conducting all of the experimental procedures with animals, sample collection, morphometric analysis of bones, biochemical evaluation of plasma and serum and statistical analysis. WK was responsible for radiological evaluation of femur with the use of quantitative computed tomography technique. BT participated in statistical evaluation of data and biochemical analyses. TS participated in the design of the study and supervised mechanical testing of bones. All authors participated in the preparation of, and have approved the final version of the manuscript.
